# Spectroscopic Characterization and Antioxidant Properties of Mandelic Acid and Its Derivatives in a Theoretical and Experimental Approach

**DOI:** 10.3390/ma15155413

**Published:** 2022-08-05

**Authors:** Monika Parcheta, Renata Świsłocka, Grzegorz Świderski, Marzena Matejczyk, Włodzimierz Lewandowski

**Affiliations:** Department of Chemistry, Biology and Biotechnology, Bialystok University of Technology, Wiejska 45E, 15-351 Bialystok, Poland

**Keywords:** mandelic acid, spectroscopy, DFT calculations, antioxidant assays, NBO

## Abstract

The following article discusses the antioxidant properties of mandelic acid and its hydroxy and methoxy derivatives. The antioxidant capacity of these compounds is determined by DPPH, FRAP, CUPRAC and ABTS. The mechanisms underlying the antioxidant properties are described by BDE, IP, PDE, ETE and PA calculation method values and referenced to experimental data. Thermochemistry, HOMO/LUMO energies, dipole moments, charge distribution, IR, RAMAN, NMR frequencies, binding lengths and angles were calculated using the B3LYP method and the 6-311++G(d,p) basis set. The structure of mandelic acid and its derivatives was determined experimentally using IR and RAMAN spectroscopy.

## 1. Introduction

### 1.1. Mandelic Acid and Its Derivatives—Properties and Applications of Studied Compounds

**Figure 1:**
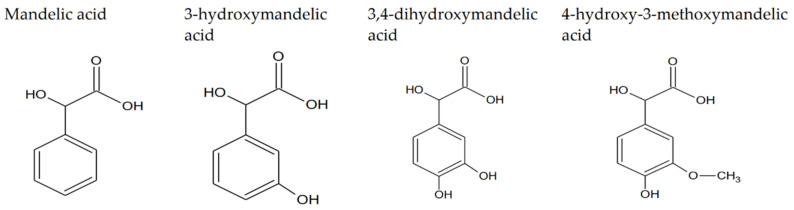
The structure of mandelic acid and its hydroxy and methoxy derivatives.

Mandelic acid (2-hydroxy-2-phenylacetic acid, MA) is a white, crystalline solid that belongs to a group of aromatic α-hydroxy carboxylic acids. The molar mass and water solubility are equal at 152.147 g/mol and 0.158 g/mL, respectively [1]. DL-mandelic acid can be derived from the hydrolysis of an extract of bitter almond [2]; it can also be isolated from *Aesculus indica* fruit [3]. Due to the presence of the chirality center in moiety, mandelic acid exists in enantiomeric forms and racemic forms [4]. Chirality underlie pharmaceutical industry applications of mandelic acid. Mandelic acid is used as a reactant in semi-synthetic penicillins, cephalosporins and antiobesity and antitumor agents production [5]. Despite its antibacterial activity, it is also used as a skincare modality agent, precursor for the pharmaceutical industry and sensing substrate for molecule recognition research [6]. Mandelic acid exhibits antibacterial properties, and due to its lack of toxic effect on organisms, it finds usage as a medicament for urinary infections and acne. The condensation reaction produces a mandelic acid condensation polymer (SAMMA). SAMMA is useful as an inhibitor of HIV, herpes viruses 1 and 2 and is active against Neisseria gonorrhoeae and Chlamydia trachomatis. It also shows activity against Gram-positive bacteria (Listeria monocytogenes and Staphylococcus aureus) and Gram-negative bacteria (Klebsiella pneumoniae and Pseudomonas aeruginosa) [7]. MA is a raw material developed in the production of polymers and rubber [8]. It also serves as a biomarker of exposure to styrene. As a biomarker, it is created in metabolic pathways and excreted through urine [9]. Triorganotin (IV) derivatives of mandelic acid have shown potent in vitro anticancer activity against mammary, liver and prostate cancers. Diorganotin (IV) derivatives of mandelic acid are more cytotoxic than triorganotin analogues [10]. The simplest derivative of mandelic acid, 3-hydroxymandelic acid, also known as *m*-hydroxymandelic acid (MHMA), is a product of the phenylephrine metabolism in the human body [11]. Midgley et al., showed that MHMA is a normal constituent of human urine [12]. Another mandelic acid derivative is 3,4-dihydroxymandelix acid, also known as DHMA. This compound can be obtained in the thermophilic reaction cascade using thermostable enzymes obtained from *Thermococus barophilus* and *Thermomonospora curvata*, of low-cost phenylpyruvic acid (PPA) and 2-phenylglyoxylic acid (PGA), as subtracts [13]. It also occurs in mammalian tissues, especially in the heart, as a decarboxylated noradrenaline metabolite [14]. Another ligand discussed in this paper is 3-methoxy-4-hydroxymandelic acid, also known as vanillymandelic acid-VMA. It can be obtained as an undesirable byproduct of the condensation reaction of glyoxalic acid and guaiacol [15]. The presence of vanillymandelic acid, produced almost exclusively in the human liver, present in human urea indicates the presence of tumor cells such as PCC, paraganglioma or neuroblastoma [16]. Since the 1970s, VMA has also been used as a biomarker of metabolic disorders such as dopamine excretion disorders, as well as neurological disorders such as autism, post-traumatic stress disorder, Parkinson’s disease or depression [17]. Mandelic acid derivatives are still poorly described in the literature. The aim of this study was to compare the structure of these compounds and their antioxidant properties in relation to mandelic acid. Discussion of the antioxidant properties of mandelic acid and its derivatives also includes the description of reaction mechanisms with free radicals and radical cations. The structures of the studied compounds are presented in Figure 1.

### 1.2. The Antioxidant Reaction Mechanisms Description

The DPPH antioxidant assay is described with different mechanisms of reaction, so it is not possible to assign one mechanism of reaction unequivocally to this assay. According to the literature, the mechanism of the reaction between the antioxidant molecule and DPPH radical depends on the used solvent. In ionizing solvents such as methanol and ethanol, DPPH reacts with phenolic compounds following the SPLET mechanism of the reaction. In ionizing solvents, this mechanism follows the HAT mechanism, which is slower than SPLET [18]. Due to the strong hydrogen atom bonding capacity of methanol solutions, electron SET (single electron transfer mechanism) is favorized over HAT (hydrogen atom transfer) [19]. The presence of acids in solution also has an influence on the mechanism of the reaction between radicals and antioxidants, e.g., hydrogen atom transfer in the HAT mechanism is inhibited in the presence of acetic acid, which suppresses the ionization of the hydrogen group of the antioxidant compound, thus proton transfer to DPPH radical is inhibited [20]. DPPH is also considered an assay with mixed mechanisms regarding HAT, SPLET, PCET (proton-coupled electron transfer) and ET-PT (electron transfer followed by proton transfer), and the mechanism of the DPPH reaction depends not only on solvent polarity but also on the structure of antioxidant and pH [21]. ABTS assay reactions are described with mixed reaction mechanisms, consisting of HAT and ET combinations [22]. FRAP and CUPRAC are both described with ET reaction mechanisms [23]. In vitro antioxidant test results are compared with thermodynamical parameters describing the mechanisms of antioxidant assays. Scavenging of radicals undergo different reaction mechanisms, among which HAT (hydrogen atom transfer) is the key reaction in biology and chemistry [24]. In the HAT mechanism, protons and electrons are simultaneously transferred from the donor to the acceptor in a one-step reaction, without any intermediate stage. This reaction does not involve significant charge distribution [25]. The HAT reaction mechanism can be described with the following equation [26]:

Ar(OH) + R^•^ → ArO^•^ + RH (1)

The HAT mechanism is described by the BDE value (bond dissociation energy) of the hydroxyl group of the antioxidant compound. The lower the BDE value is, the higher the ability of the hydrogen abstraction grom hydroxyl group, and the higher the antioxidant capacity of the compound. HAT does not involve charge separation; hence this mechanism is favorable in a non-polar environment [27]. Below, the equation for BDE value calculation is presented [28].

BDE = H_(ArO_^•^_)_ + H_(H)_ − H_(ArOH)_ (2)

where H_(ArO_^•^_)_ is the enthalpy of aromatic radical formation in the reaction of hydrogen abstraction, H_(H)_ is the enthalpy of the hydrogen atom and H_(ArOH)_ is the enthalpy of the neutral molecule.

Another mechanism involved in providing antioxidant capacity is the sequential proton loss electron transfer (SPLET) mechanism. This reaction can be described by the following reactions [29]:

Ar(OH) → ArO^−^ + H^+^ (3)

ArO^−^ +R^•^ → ArO^•^ + R^−^ (4)

R^−^ + H^+^ → RH. (5)

The SPLET mechanism is described with PA (the proton affinity) and the electron transfer enthalpy (ETE). Values of these thermochemistry parameters can be obtained according to Equations (6) and (7), respectively.

PA = H(ArO^−^) + H(H+) − H(ArOH) (6)

ETE = H_(ArO_•_)_ + H_(e_^−^_)_ − H_(ArO_^−^_)_ (7)

where H_(ArO_−_)_ is the enthalpy of the aromatic anion, H_(H_+_)_ is enthalpy of the proton and H_(e_−_)_ is the enthalpy of the electron. 

The last mechanism underlying antioxidant properties is single electron transfer followed by proton transfer (SET-PT). This mechanism is described with reactions (8) and (9):

ArOH + R^•^ → ArO^+•^ + R^−^ (8)

ArO^+^• + R^−^ →RH + ArO•. (9)

The SET-PT mechanism is described by the proton dissociation enthalpy (PDE), according to Equation (10) and the ionization potential (IP) provided as Equation (11) [29].

PDE = H(ArO•) + H(H^+^) − H(ArO^+^•) (10)

where H(ArO^+^•) is the enthalpy of the antioxidant radical cation.

IP = H(ArO^+^•) + H(e-) − H(ArOH). (11)

All the transfer processes cited above are known as proton-coupled electron transfer (PCET) [30].

## 2. Materials and Methods

### 2.1. Materials

Mandelic acid 98%, 3-hydroxymandelic acid ≥ 97% and 3,4-dihydroxymandelic acid 95% were purchased from Sigma Aldrich (Saint Louis, MO, USA), 4-hydroxy-3methoxymandelic acid 98% was purchased from Alfa Aesor (Kandel, Germany), CuCl_2_ × 2H_2_O, FeCl_3_ × 6H_2_O, ABTS (2,2′-azino-bis(3-ethylbenzothiazoline-6-sulfonic acid), Trolox and H_2_O_2_, were purchased from Sigma Aldrich and used without purification. KBr, DPPH (2,2-diphenyl-1-picrylhydrazyl), TPTZ (2,4,6—trypyridyl-s-tirazine), FeCl_3_, FeSO_4_ × 7H_2_O, neocuproine and ammonium acetate were purchased from Sigma Aldrich. Hydrochloric acid (35%), methanol and ethanol (analytical grade) were purchased from Chempur (Poland).

### 2.2. FTIR and Raman Spectra

The Ft-IR spectra were registered in KBr matrix pellets on an Alfa Bruker spectrometer (Bremen, Germany) within the range of 400–4000 cm^−1^ with a resolution of 4 cm^−1^. FT-Raman spectra of solid samples were recorded with a MultiRam (Bruker, Bremen, Germany) spectrometer in the range of 400–4000 cm^−1^.

### 2.3. NMR Spectra

1H NMR and 13C NMR spectra of the DMSO samples solution of the studied compound were recorded with a Bruker Avance II 400 MHz unit at room temperature with TMS as an internal reference

### 2.4. Evaluation of Antioxidant Activity

In this paper, the antioxidant properties of the tested compounds were evaluated using DPPH, ABTS, FRAP and CUPRAC assays. The antioxidant capacity of compounds can be measured using stable and intensely colored radical 2,2-diphenyl-1-picrylhydrazyl (DPPH). The DPPH assay consists of measuring the ability of antioxidant compounds to quench DPPH radicals, expressed as a percentage of DPPH^•^ turned into hydrazine DPPH-H form. The reaction of reducing the unpaired electrons of nitrogen atoms in DPPH^•^ is visualized by a change in color of the examined solution from violet to yellow [31]; hence the antioxidant capacity of antioxidant can be measured using the spectrophotometric method as a decrease in absorbance value at about 515–520 nm [32]. H–atom donation by an antioxidant molecule to DPPH^•^ can be described using a single electron transfer or hydrogen atom transfer mechanism, depending on the reaction environment [33]. The initial water and ethanolic solutions were prepared at concentrations of 0.25 µM–5 µM for the investigated mandelic acids. The methanolic solution of DPPH was prepared at a concentration of 15 µM. Tested compounds were prepared in testing tubes, where the appropriate dilutions were made to obtain the abovementioned scope of concentrations, with the final volume after dilution equal to 1 mL for water and ethanol series separately. Then, 2 mL of DPPH solution were added to each testing tube. All samples were incubated in darkness for an hour. The absorbance of the samples was measured at 516 nm against water and ethanol as a blank for water and ethanol series, respectively, using an Agilent Carry 5000 spectrophotometer (Santa Clara, CA, USA). Since DPPH is not highly soluble in hydrophobic solvents, to determine antioxidant capacity in organic media, the 2,2’-azinobis(3-ethylbenzothiazoline-6-sulfonate) radical cation (ABTS^•+^) can be applied. Apart from good solubility in organic solvents, the ABTS assay can be implemented over a wide range of pH values [34]. Green radical cation ABTS^•+^ chromophore formed in the reaction of ABTS with potassium persulfate is reduced by an antioxidant to an extent depending on the concentration of the antioxidant and the duration of the reaction. The total antioxidant activity of the compound in the ABTS assay is designated using the spectrophotometric method [35]. ABTS water solution was prepared at a concentration of 5.4 mM and mixed with potassium persulfate at a concentration of 1.74 mM at a 1:1 ratio. After 12 h of incubation, the solution was diluted with methanol to achieve an absorbance value between 0.7 and 0.8 at 734 nm. The scope of the concentrations of the tested compounds was the same as at the DPPH assay, but dilutions were made to achieve a final volume of 1.5 mL for both water and ethanolic series. To prepare the concentration scope of water and ethanolic solutions of mandelic acids compounds in this way, 1.5 mL of ABTS solution were added. The absorbance value was measured at 734 nm against 1.5 mL of appropriate solvents mixed with 1.5 mL of ABTS solution as a blank. Results of both the DPPH and ABTS assays are expressed as IC_50_ values, corresponding to the concentration of antioxidant that is required to decrease the initial concentration of DPPH radical or ABTS radical cations by 50% [36]. The % of inhibition in DPPH and ABTS assays was calculated using the following equation:

$$\%\;I=\frac{A_{control}-A_{sample}}{A_{control}}$$

where % I is the % of inhibition of DPPH or ABTS radical, $A_{control}$ is the absorbance of the control and $A_{sample}$ is the absorbance of the sample. 

IC_50_ was designated as the dependence between the concentrations of tested compounds and their % I values. Another antioxidant capacity assay is CUPRAC (cupric reducing antioxidant capacity). In this method, the working solution contains CuCl_2_ × 2 H_2_O (0.01 M), neocuproine (0.0075 M) and ammonium acetate at pH 7 (1.07 M), mixed at a 1:1:1 ratio. Absorbance values are measured at 450 nm [37] using an Agilent Carry 5000 spectrometer. To the 3 mL of CUPRAC solution, 0.5 mL of tested compound (at 1 mM for mandelic acid and 3-hydroxymanelic acid, 0.1 mM for 4-hydroxy-3-methoxymandelic acid and 3,4-dihydroxymandelic acid) and 0.6 mL of distillated water were added. The CUPRAC assay consists of the creation of a copper (II)–neocuproine complex, which is reduced by an antioxidant compound to a colored copper(I)–neocuproine chelate complex. This method is used in both oil and water solutions [38]. Antioxidant activity was expressed as the Trolox equivalents [µM], using the calibration curve prepared over the range of 0.05–0.35 mM. FRAP (Ferric reducing activity power assay) evaluates total antioxidant activity in the reaction of reducing the ferric tripyridyl triazine (Fe(III) TPTZ) complex to blue ferrous tripyridyl triazine (Fe(II)-TPTZ) form at low pH, in the presence of an antioxidant, which can be monitored by measuring the change in absorption at 593 nm [39]. To prepare the FRAP reagent acetate buffer (300 mM), TPTZ (10 mM) and FeCl_3_ (20 mM) were mixed together at a 10:1:1 ratio. Next, 0.4 mL of each of the tested compounds at the same concentrations as in the CUPRAC assay were mixed with 3 mL of the FRAP reagent. Then, samples were incubated in darkness for 8 min. Antioxidant activity was expressed as Fe^2+^ equivalents [µM], using the calibration curve prepared over the range of 0.05–0.3 mM. All measurements in the performed antioxidant assays were taken in two series of five repetitions for every compound and every concentration in two different solutions. 

### 2.5. Computational Details

All calculations were performed using the Gaussian09 program package. Optimization and geometries of the studied mandelic acids and corresponding radicals, anions and radical cations were calculated using the B3LYP method and the 6-311++G(d,p) basis set. All calculations were performed for vacuum and for water and ethanol solvents using the same method and basis set. Enthalpies for BDE, IP, PDE, PA and ETE were calculated for 298.15 K and 1.0 atmospheric pressure. The calculated gas-phase enthalpy for proton, electron and hydrogen atoms were taken from the literature and were equal to 6.197 kJ/mol, 3.146 kJ/mol [40] and −1306 kJ/mol, respectively [41]. The solvent phase calculations in the water of the proton, electron and hydrogen atoms were, respectively: −1058 kJ/mol [40], −101 kJ/mol and −4 kJ/mol [41]. Additionally, for the solvent phase in ethanol, these values were equal to −1068.4 kJ/mol, −73.6 kJ/mol [40] and −3.7 kJ/mol for hydrogen atoms [41]. The HOMO and LUMO energies were calculated. HOMA aromaticity indices for vacuum, water and ethanol were calculated. Parameters such as softness, hardness, electronegativity and electrophilicity were also calculated. 

The aromaticity of studied acids was designated as the HOMA index on the basis of the following Formula [42]:

$$\mathrm{HOMA}=1\;-\;[\alpha (R_{\mathrm{opt}}\;–\;R_{\mathrm{ar}}\;{)}^{2}+\frac{\alpha }{n}\;\sum (R_{\mathrm{ar}}-R_{i}{)}^{2}]=1\;–\;\mathrm{EN}-\mathrm{GEO}$$

where:

R_opt_ is the optimal value of a bond length. For C-C type of bonds in a benzene ring, the R_opt_ value is equal to 1.334;
R_i_ is the length of the ith bond;
n is the number of bond lengths in the ring;
R_ar_ is the average bond length;
α is the normalization factor necessary to obtain a HOMA value equal to 1 for ideally aromatic benzene or 0 for an ideally alternating cyclohexatriene Kekulé ring.

In this study, I_6_ and BAC aromaticity indexes are also calculated. The I_6_ aromaticity index, also known as Bird’s aromaticity index, is defined as:

$$I_{6}\;=100\;(1-\frac{V}{33.3})\;$$

where

$$V=\frac{100}{N_{av}}\sum {\left(N_{N}-N_{av}\right)}^{2}$$

*N* is the bonds order provided by (a/R^2^ − b), where a and b are empirical constants, and R is the bond length [43]. Another approach to determining the aromaticity is the so-called bond alteration coefficient BAC, defined as BAC = $\underset{r}{\sum }(R_{r\;}-R_{r+1}$), where R_r_ and R_r+1_ are consecutive bond lengths in the ring [44].

## 3. Results

### 3.1. The Antioxidant Activity of Mandelic Acid and Its Derivatives

**Figure 2:**
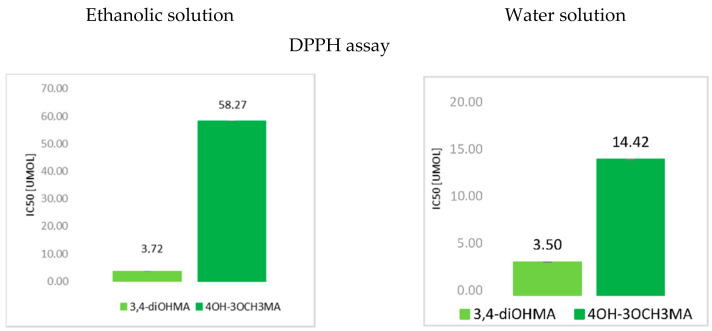

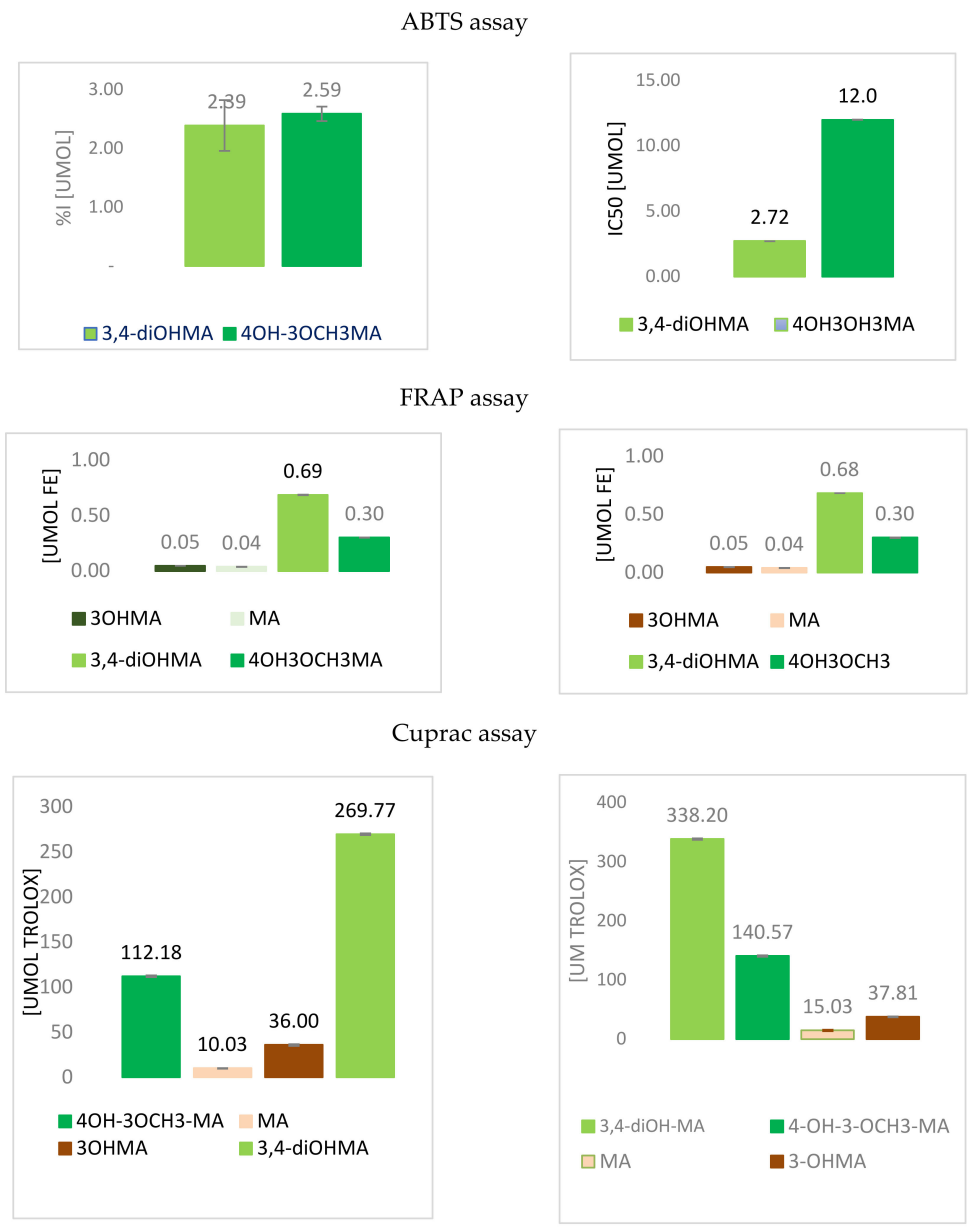
Comparison of antioxidant activities of mandelic acid and its derivatives in water in ethanolic solutions using DPPH, ABTS, FRAP and CUPRAC assays.

The lower the value of IC_50_ in DPPH and ABTS assays, the better antioxidant properties of the tested compounds. The higher FRAP and CUPRAC values, the higher ferric and cupric reducing activities of these compounds (Figure 2). Thus, according to the DPPH and ABTS assays, 4-hydroxy-3-metoxymandelic acid exhibits weaker antioxidant properties than 3,4-di-hydroxymandelic acid. 3-hydroxymandelic acid and mandelic acid did not exhibit antiradical activity in these assays. According to the FRAP and CUPRAC assays, the antioxidant properties of the tested compounds grew as follows: 3-hydroxymandelic acid < mandelic acid < 4-hydroxy-3-metoksymandelic acid < 3,4-dihydroxymandelic acid.

### 3.2. Computational Results

#### 3.2.1. Structure of Mandelic Acid and Their Derivatives

**Figure 3:**
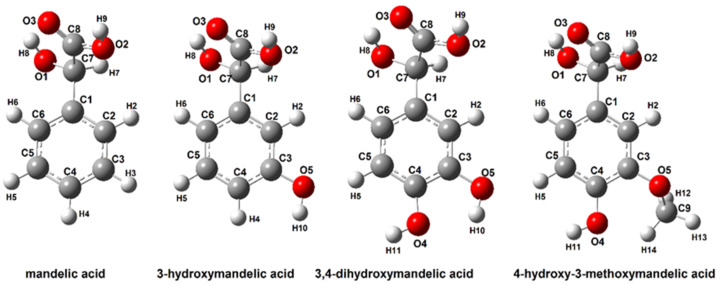
Optimized structures of mandelic acid and their derivatives calculated in B3LYP/6-311++G(d,p).

The structures of mandelic acid and its derivatives were optimized by the B3LYP/6-311++G (d, p) method. Calculations of the NBO electron charge distribution, thermodynamic parameters, theoretical NMR and IR spectra and energy of HOMO and LUMO molecular orbitals were performed for optimized conformer structures of modeled molecules. Table 1 show the energy values and dipole moments of the optimized structures. Figure 3 show the optimized molecules with atom numbering used for the description of NMR, NBO and other parameters.

#### 3.2.2. Bond Dissociation Enthalpy, Ionization Potentials, Proton Dissociation Enthalpies, Proton Affinities and Electron Transfer Enthalpies for Mandelic Acid and Its Derivatives

BDE, IP, PDA, PA and ETE were calculated using the abovementioned equations. In Table 2, the results are presented.

The BDE energy value parameter describes the ability to donate H atoms. The minimum BDE value indicates the greatest possibility of hydrogen abstraction in the substituent, thus which substituent is the most susceptible to radical attack [30]. The calculations of thermodynamical parameters related to the reactivity of the studied compounds (in relation to free radical) showed that substituted aromatic ring position derivatives of mandelic acid require less energy expenditure in reactions related to the antioxidant activity of these compounds. The values of the energy of the dissociation process (BDE) are the lowest for 4-hydroxy-3-methoxymandelic acid. In reactions with free radicals, mandelic acid requires the highest energy expenditure among the other tested compounds.

#### 3.2.3. Aromaticity

For the optimized mandelic acid structures (Figure 3) (calculated in gas, water and ethanolic phase with B3LYP-6-311++G(d,p)) method), the aromaticity indices were calculated. Three calculation models (HOMA, I6 and BAC indices) based on the bond lengths in aromatic rings, were used. The calculation results are presented in Table 3.

The Aromaticity indices of hydroxy and methoxy derivatives of mandelic acid are lower than those of pure mandelic acid. The aromaticity of the π-electron aromatic ring is reduced by the attachment of one hydroxyl group, two hydroxyl groups or both hydroxyl groups simultaneously. It is consistent with the calculated aromaticity indices in each of the tested solvents (water and ethanol) and in the aqueous phase. The attachment of two hydroxyl groups to the aromatic ring causes a greater decrease in aromaticity values than the attachment of one hydroxyl group. An even greater decrease in aromaticity is observed when a methoxy group in the aromatic ring of the hydroxymandelic acid is substituted for the second hydroxyl group. 4-hydroxy-3-methoximandelic acid is characterized by the lowest aromaticity. Substitution of the methoxy group causes a greater increase in the disruption of the π-electron system in the aromatic ring than in the hydroxyl group. The aromaticity of the studied compounds changes in series: Mandelic acid > 3-hydroxymandelic acid > 3,4-dihydroxymandelic acid > 4-hydroxy-3-methoxy mandelic acid.

#### 3.2.4. HOMO and LUMO Parameters

**Figure 4:**
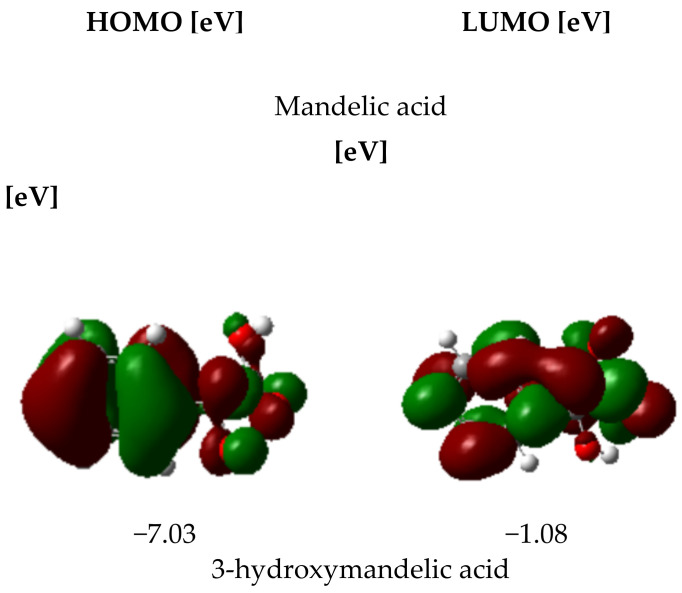

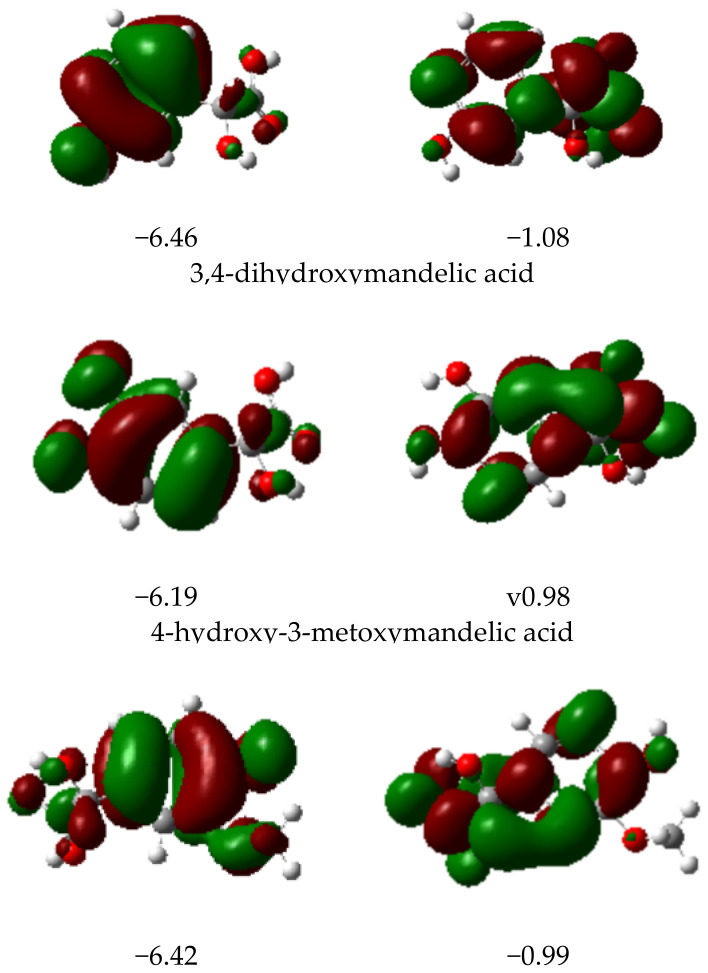
HOMO and LUMO energies [eV] distribution in mandelic acid and its derivatives in vacuum calculated at B3LYP/6-311++G (d,p) level of theory.

For every tested compound, HOMO and LUMO energies in vacuum, ethanol and water were calculated, and then other electronic parameters such as energy gap, electroaffinity, electronegativity, chemical hardness and softness were designated. Below, in Figure 4, the HOMO and LUMO energies are presented.

The designation of HOMO and LUMO parameters is a very helpful tool in the provision of antioxidant properties of tested compounds. The HOMO orbital energy value describes the electron-donating properties of the molecule. The higher the HOMO value, the better the antiradical properties of the compound. The ionization potential provides information regarding the electron removing facility. The lower the ionization potential, the lower the energy required to remove an electron. The reactiveness and stability of compounds can be predicted by assessing the difference value between HOMO orbital energy and LUMO orbital energy (ΔE). The higher the value of that difference, the lower the reactivity and stability of the compounds. In Table 4, the values of the calculated electronic parameters are provided. According to the HOMO and LUMO energy values, the mandelic acid derivatives analyzed in the frame of that work are characterized by lower antioxidant activity than mandelic acid. The ΔE parameter shows that the difference in HOMO and LUMO energy levels is reduced due to the substitution of the aromatic ring with –OH and –OCH_3_ functional groups, which leads to the distribution of electronic charge in the aromatic ring. Further analyses such as the NBO electron charge distribution and EPS electrostatic potential distribution maps provide information on the reactivity of individual fragments of the studied molecules.

#### 3.2.5. Electron Charge Distribution and EPS Distribution

The electron charge distribution calculated by the Natural Bond Orbital method for the structures optimized with the B3LYP/6-311++G(d,p) method of the investigated mandelic acid derivatives is presented in Table 5. The calculations were performed for two solvents and structures optimized in the gas phase. The analysis of changes in the distribution of electronic charge, with particular emphasis on the aromatic ring, showed that the electronic system of the aromatic ring in the mandelic acid molecule is disturbed (aromaticity decrease) due to the substitution of hydroxyl/methoxy groups in this aromatic ring. The electronegative oxygen atoms substituted in the aromatic ring shift the electron cloud of the aromatic ring towards the substituents, which changes the reactivity of the aromatic ring. The electron charge values of NBO on the aliphatic carbon atoms designated as C7 and C8 in mandelic acid derivative structures remain the same in relation to the atomic charge value in mandelic acid. The greatest changes in the charge distribution of NBO are observed at the carbon atoms substituted in the C3 and C4 positions. In the case of monosubstituted hydroxymandelic acid, a slight increase in the electronic charge distribution around the C3 and C4 atoms is observed, while in the case of disubstituted mandelic acid derivatives, e.g., 3,4-dihydroxymandelic acid and 3-methoxy-4-hydroxy mandelic acid, values of the electron charge decrease significantly. The distribution of electronic charge in the aromatic ring of the mandelic acid molecule substituted with one or two hydroxyl groups (or hydroxyl and methoxy group) demonstrates the reduced aromaticity of these ligands compared to the ligand molecule unsubstituted in the aromatic ring. The results are consistent with the observations made with the calculated aromaticity indices. The NBO electron charge distribution does not change significantly due to the substitution of the aromatic ring of the mandelic acid molecule.

**Figure 5:**
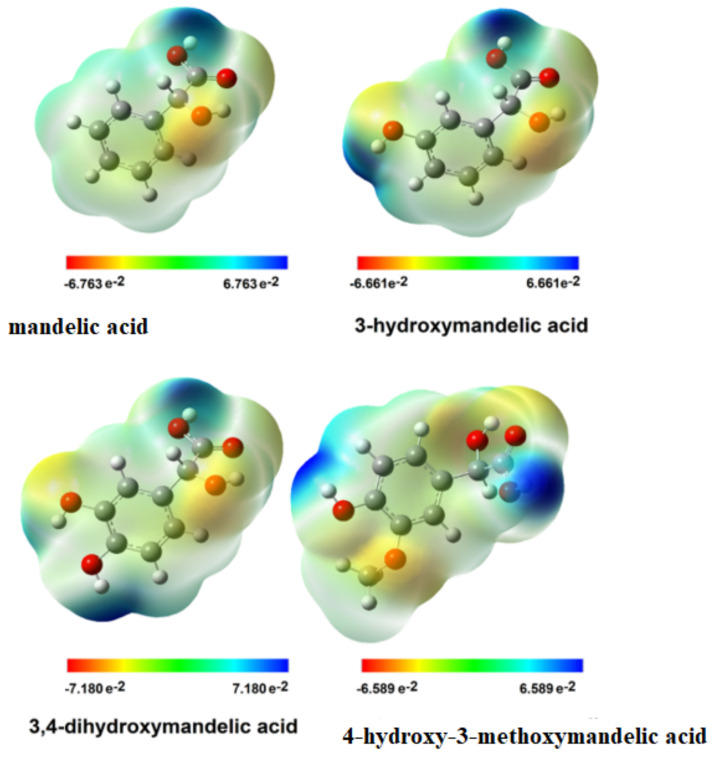
Maps of the electrostatic potential distribution of EPS in mandelic acid and its derivatives.

The electrostatic potential map shows the areas of a molecule related to its electrophilic (red) and nucleophilic (blue) reactivity (Figure 5).

The Electrostatic potential map shows the regions of the molecules related to their electrophilic (red) and nucleophilic (blue) reactivity (Figure 5). In mandelic acid, 3-hydroxy mandelic acid and 3,4-dihydroxymandelic acid, the hydroxyl group of the carboxylic moiety is susceptible to nucleophilic attack which cannot be observed in 4-hydroxy-3-methoxy mandelic acid. In the case of the latter molecule, the methoxy group is susceptible to nucleophilic attack. In the studied molecules, the hydrogen atoms in the aromatic ring are susceptible to nucleophilic substitution. The protons of these groups are electron-poor due to the shift of the electron cloud towards electronegative oxygen atoms, making them susceptible to the nucleophilic attack, which affects the reactivity of these groups in reaction with free radicals having an unpaired electron. Increasing the amount of hydroxyl (methoxy) groups contributes to the greater reactivity of these molecules in the reaction with free radicals. The maps of the distribution of electrostatic charges show that mandelic acid is the least susceptible to attack by free radicals because it has only one nucleophilic center, the carboxylic acid proton.

### 3.3. FT-IR and Raman Spectroscopy

The characteristic bands of the carbonyl stretching vibrations appear in both the IR and Raman spectra of mandelic acid and its derivatives (Table 6). In the IR spectra of mandelic acid, this band is placed at 1716 cm^−1^. In a similar placement, the stretching band ν_C=O_ in 3-hydroxymandelic acid occurred (1715 cm^−1^). The greatest shift in the localization of these bands is observed in 34-dihydroxymandelic acid and 4-hydroxy-3-methoxymandelic acid. In the case of 3,4-dihydroxymandelic acid, this band is shifted toward a lower wavenumber (1708 cm^−1^) compared to mandelic acid, and in the IR spectra of 4-hydroxy-3-methoxymandelic acid, this band is shifted towards a higher wavenumber (1743 cm^−1^). The bands assigned to the stretching vibrations between aromatic carbon atoms are shifted toward growing wavenumbers in the series: 3-hydroxymandelic acid (1466 cm^−1^) → mandelic acid (1497 cm^−1^) → 4-hydroxy-3-methoxymandelic acid (1517 cm^−1^) → 3,4-dihydroxymandelic acid (1537 cm^−1^). In the Raman spectra, the band originating from the stretching vibrations of the carbonyl group in mandelic acid occurred at 1719 cm^−1^, in 4-hydroxy-3-methoxymandelic acid at 1716 cm^−1^. In 3,4-dihydroxymandelic acid, this band is shifted to the lower wavenumber and occurred at 1648 cm^−1^, whereas in the spectra of 3-hydroxymandelic acid, this band did not occur. In the Raman spectra, the bands derived from the stretching bonds ν_CC_ in aromatic rings appeared only in mandelic acid at 1588 cm^−1^ and in 3,4-dihydroxymandelic acid at 1605 cm^−1^. In the IR and Raman spectra of mandelic acid derivatives, the bands derived from hydroxyl and methoxy groups also appeared. In 3,4-dihydroxymandelic acid and 4-hydroxy-3-methoxymandelic acid, the bands of stretching vibrations of aromatic hydroxyl groups appeared at 3420 cm^−1^ and 3402 cm^−1^, respectively. This band is absent in the Raman spectra of 4-hydroxy-3-methoxymandelic acid, but the stretching bands of the methoxy group appeared at 954 cm^−1^ in the IR spectra and at 953 cm^−1^ in the Raman spectra. The location of aromatic bands, related to the vibrations of the π-electron system, determines the influence of the substituents on electron charge distribution in the molecule. The decrease in intensity, the fading of the bands or the shift toward lower wavenumbers indicates the disturbance in the charge distribution in the ligand’s aromatic ring. In the case of bonds in the aromatic ring system (19a, 19b, 8b, 9a, 18b,18a), the spectra of the mandelic acid derivatives showed higher wavenumbers than the corresponding bands in mandelic acid. It was also observed that a number of bands in the mandelic acid spectrum (e.g., 16a, 16b, 17a,9b) shifted towards lower wavenumbers, demonstrating that the substitution of mandelic acid with the hydroxyl/methoxy group in the aromatic ring increases the aromaticity disturbance of the ligand.

#### NMR Study

^13^CNMR and ^1^HNMR chemical shifts for mandelic acid and their derivatives are presented in Table 7.

***^13^C NMR***

Substitution of the aromatic ring with a hydroxyl group, two hydroxyl groups or with a methoxy group in the 3- and 4-position in the mandelic acid molecule does not change the electron density of carbon atoms marked as C7 and C8 (Figure 1). A slight chemical shift of these atoms can only be noticed when comparing the ^13^C NMR spectrum with the spectra of hydroxy and methoxy derivatives of mandelic acid. Substitution with a hydroxyl group in the 3-position of the aromatic ring of mandelic acid causes a slight decrease in electron density around the C1 and C6 carbon atoms, which can be observed in the ^13^C NMR spectrum of 3-hydroxymandelic acid as a shift towards higher chemical shift values, while substitution with another hydroxyl or methoxy group increases electron density (change in the value of chemical shifts in the reverse direction). The C2 carbon chemical shift in substituted mandelic acids is significantly lower than in the ligand deprived substituents in the aromatic ring. It proves the increase of electron density around the C2 atom of mandelic acid after introducing the substituents. The introduction of substituents to the aromatic ring of mandelic acid causes significant changes in the electron density around the atoms to which the substituents are attached. Around the 3C atom in 3-hydroxymandelic acid, there is a significant decrease in electron density (a significant increase in the value of chemical shifts in the ^13^C NMR spectra) in relation to the mandelic acid. Substituting another substituent, the change in density around 3C in disubstituted acids versus mandelic acid is slightly less due to the attraction of the electron cloud by the second substituent. Changes in the electron charge distribution (charge distribution disturbance) in substituted mandelic acid derivatives in relation to the unsubstituted ligand affect its reactivity. The aromaticity of these systems is lower than that of mandelic acid.

***^1^H NMR***

The calculated aromaticity indexes indicate that the most stable electron system of the aromatic ring is found in mandelic acid. Substitution with a hydroxyl group, two hydroxyl groups and a methoxy group reduces the aromaticity of mandelic acid. Proton chemical shifts in the ^1^H NMR spectra show the same effect. A decrease in the value of aromatic proton shifts (2a, 5a and 6a) were observed in hydroxymandelic acid, 3,4,-dihydroxymandelic acid and 4-hydroxy-3-methoxymandelic acid. A decrease in the value of the chemical shift of the aliphatic proton 7a was also observed, while the value of the chemical shifts of 8a proton increased in the mandelic acid derivatives substituted in the aromatic ring. The experimentally determined values of the chemical shift of aromatic and aliphatic protons are similar to the theoretical calculations determined by the GIAO method for optimized structures by the B3LYP/6-311++G(d,p) method using the DMSO solvent model. Significant differences between the experimental and theoretical values of chemical shifts of hydroxyl and carboxyl protons probably result from the influence of hydrogen interactions in the structure of tested compounds, which does not occur in the calculated monomers.

## 4. Conclusions

The study of the electronic structure of 3-hydroxymandelic acid, 3,4-dihydroxymandelic acid and 4-hydroxy-3-methoxymandelic acid showed that the substitution of the aromatic ring with a hydroxyl group/groups and methoxy group changed the π-electron system in the mandelic acid structure. The analyses were carried out by several experimental (FTIR, FT-Raman and NMR) and quantum theoretical methods (calculations of the structure, aromaticity and NBO electronic charge distribution). The results of the experimental and theoretical methods are consistent. Substitution in the aromatic ring of mandelic acid with substituents containing an electronegative oxygen atom increases the disturbance of the electronic system of the aromatic ring (decrease in aromaticity) of this acid. The decrease in ring aromaticity increases the reactivity of the molecules, which is consistent with the theoretical calculations of the HOMO and LUMO energies as well as other descriptors such as electro-affinity and calculated reactivity parameters such as bond dissociation enthalpies, ionization potential, proton dissociation enthalpies, proton affinity and electron transfer enthalpies. The presence of hydroxyl and methoxy groups in the aromatic ring of carboxylic acids may influence their reactivity towards free radicals, which was also investigated. The antioxidant activity of the studied compounds was tested using DPPH and ABTS radicals and the reduction abilities in the FRAP and CUPRAC assays. The conducted research shows that 3-hydroxymandelic acid, like unsubstituted mandelic acid, shows very weak antioxidant properties in ABTS and DPPH assays. The best antioxidant properties are demonstrated by 3,4-dihydroxymandelic acid, and slightly weaker antioxidant properties are shown by 4-hydroxy-3-methoxymandelic acid. Additionally, 3,4-dihydroxymandelic acid presented the highest reduction potential in FRAP and DPPH assays. The presence of hydroxyl and methoxy substituents changes the antioxidant potential of mandelic acid, while the presence of two hydroxyl groups has a greater effect than the introduction of a methoxy group. One hydroxyl group substituted in the aromatic ring does not significantly increase the reduction potential of mandelic acid or its antioxidant activity. The change in electron charge distribution in the aromatic ring of mandelic acid caused by substitution increases the reactivity of this acid, including its antioxidant potential. For the structures optimized by the B3LYP/6-311++G(d,p) method, electron potential maps were carried out with the EPS method. EPS maps show that hydroxyl groups are reactive centers, susceptible to attack by nucleophile molecules (which are free radicals having one unpaired electron). The methoxy group is less susceptible to attack by nucleophilic molecules. In the model system calculated theoretically by the DFT method (B3LYP/6-311++G(d,p)), the influence of the solvent on the analyzed molecules was compared with the experimental results and calculations performed in the gas phase. The solvent does not significantly affect the results of the calculations; in particular, it affects the calculations of the structure and electron charge distribution of the studied molecules. The calculations of the thermodynamic parameters related to the reactivity of the tested compounds (in relation to free radicals) show that the substituted mandelic acid derivatives require less energy expenditure in the reactions related to the antioxidant activity of these compounds.

## Figures and Tables

**Table 1:** Structural parameters of studied mandelic acid and its derivatives.

	Mandelic Acid	3-Hydroxymanndelic Acid	*3,4-Dihydroxymandelic Acid*	*4-Hydroxy-3-Metoxymandelic Acid*
*Energy [hartree]*	*−535.51*	*−610.76*	*−685.99*	*−725.31*
*Energy [kJ/mol]*	−1,406,909.87	*−1,604,604.26*	−1,802,266.09	−1,905,551.83
*Dipole moment [De]*	*2.41*	*1.92*	*0.27*	*1.47*

**Table 2:** Thermodynamical parameters of mandelic acid and its derivatives in vacuum, water and ethanolic solutions obtained at the B3LYP/6-311++G(d,p) level of theory.

BDE [kcal/mol]				
	Compound	Vacuum	Water	Ethanol
Solvent				
**MA**		97.65	407.23	409.11
**3OH-MA**		77.06	396.1	389.21
**3,4-diOH-MA**				
**3-OH radical**		70.74	382.56	384.45
**4-OH radical**		62.21	382.45	384.33
**4OH-3OCH_3_-MA**		69.9	382.16	383.9
**IP [kcal/mol]**				
**MA**		199.33	131.92	139.63
**3OH-MA**		188.75	136.01	142.10
**3,4-diOH-MA**		178.05	113.81	121.47
**4OH-3OCH_3_-MA**		176.92	112.78	120.36
**PDE [kcal/mol]**				
**MA**		212.48	0.06	−4.18
**3OH-MA**		202.49	−15.16	−13.31
**3,4-diOH-MA**				
**3-OH radical**		206.85	−6.49	−10.67
**4-OH radical**		198.32	−6.60	−10.78
**4OH-3OCH_3_-MA**		207.14	−5.86	−10.10
**PA [kcal/mol]**				
**MA**		318.21	42.12	40.92
**3OH-MA**		331.68	30.38	28.85
**3,4-diOH-MA**				
**3-OH radical**		329.34	29.84	28.28
**4-OH radical**		315.39	23.25	21.30
**4OH-3OCH_3_-MA**		326.11	28.64	26.99
**ETE [kcal/mol]**				
**MA**		93.60	89.86	94.55
**3OH-MA**		59.55	90.47	86.71
**3,4-diOH-MA**				
**3-OH radical**		55.56	77.47	82.53
**4-OH radical**		61.01	83.96	89.39

**Table 3:** Aromaticity indices (HOMA, Bird’s indices (I6) and BAC) for mandelic acid and its derivatives for gas phase, water and ethanolic solutions.

Aromaticity Indice	Solution/Gas Phase	Mandelic Acid	3-Hydroxy- Mandelic Acid	3,4-Dihydroxy- Mandelic Acid	4-Hydroxy-3-Methoxy Mandelic Acid
HOMA	Water	0.989	0.988	0.980	0.986
	Gas phase	0.989	0.988	0.988	0.984
	Ethanol	0.984	0.989	0.980	0.980
I6	Water	98.90	97.74	96.57	95.84
	Gas phase	98.90	97.74	97.75	95.81
	Ethanol	98.53	98.65	96.53	95.84
BAC	Water	0.984	0.959	0.940	0.930
	Gas phase	0.984	0.959	0.959	0.918
	Ethanol	0.975	0.980	0.939	0.930

**Table 4:** Values of electronic parameters of studied ligands at the B3LYP/6-311 ++ G (d,p) level.

Mandelic Acid					
Solvent	ΔE [eV]	Hardness [eV]	Softness [eV]	Electrophilicity [eV]	Electronegativity [eV]
Ethanol	0.223	0.112	8.965	0.102	0.151
Water	0.223	0.112	8.954	0.102	0.151
Vacuum	0.218	0.109	9.154	0.050	0.149
**3-hydroxymandelic acid**					
Ethanol	0.203	0.102	9.830	0.097	0.140
Water	0.204	0.102	9.814	0.097	0.140
Vacuum	0.198	0.099	10.120	0.097	0.139
**3,4-dihydroxymandelic acid**					
Ethanol	0.190	0.100	10.406	0.090	0.133
Water	0.193	0.100	10.388	0.090	0.133
Vacuum	0.191	0.100	10.449	0.090	1.132
**4-hydroxy-3-methoxymandelic acid**					
Ethanol	0.200	0.100	10.004	0.090	0.137
Water	0.201	0.100	9.970	0.137	0.137
Vacuum	0.200	0.100	10.016	0.090	0.136

**Table 5:** NBO Atom Charge Distribution for mandelic acid and its hydroxy end methoxy derivatives.

NBO Atom Charge Distribution			
Mandelic Acid			
Atom *	Ethanol	Water	Vacuum
C1	−0.063	−0.064	−0.060
C2	−0.196	−0.198	−0.192
C3	−0.202	−0.201	−0.197
C4	−0.205	−0.205	−0.200
C5	0.201	−0.202	−0.194
C6	−0.108	−0.197	0.190
C7	0.030	0.029	0.036
C8	0.810	0.810	0.799
H2	0.215	0.219	0.207
H3	0.215	0.215	0.205
H4	0.214	0.215	0.205
H5	0.214	0.215	0.206
H6	0.218	0.215	0.220
H7	0.212	0.212	0.201
H8	0.487	0.487	0.481
H9	0.585	0.506	0.488
O1	−0.750	−0.751	−0.729
O2	−0.670	−0.670	−0.671
O3	−0.636	−0.637	−0.613
**3-hydroxymandelic acid**			
C1	−0.042	−0.043	−0.04
C2	−0.273	−0.273	−0.273
C3	0.319	0.318	−0.323
C4	−0.257	−0.258	−0.250
C5	−0.181	−0.182	−0.176
C6	−0.229	−0.230	−0.226
C7	0.031	0.030	0.037
C8	0.810	0.810	0.800
H2	0.223	0.223	0.218
H4	0.223	0.223	0.218
H5	0.216	0.216	0.206
H6	0.216	0.217	0.209
H7	0.213	0.213	0.203
H8	0.487	0.488	0.481
H9	0.506	0.506	0.488
H10	0.487	0.488	0.468
O1	−0.758	−0.751	−0.732
O2	−0.669	−0.669	−0.669
O3	−0.636	−0.637	−0.613
O5	−0.692	−0.692	−0.672
**3,4-dihydroxymandelic acid**			
C1	−0.073	−0.074	−0.081
C2	−0.253	−0.253	−0.213
C3	0.274	0.273	0.254
C4	0.271	0.271	0.283
C5	−0.257	−0.257	−0.265
C6	−0.208	−0.208	−0.178
C7	0.032	0.031	0.039
C8	0.809	0.818	0.799
H2	0.218	0.219	0.217
H5	0.219	0.220	0.284
H6	0.220	0.220	0.221
H7	0.211	0.212	0.200
H8	0.486	0.497	0.481
H9	0.505	0.505	0.488
H10	0.488	0.489	0.470
H11	0.488	0.489	0.466
O1	−0.751	−0.752	−0.730
O2	−0.671	−0.671	−0.671
O3	−0.638	−0.639	−0.613
O4	−0.686	−0.687	−0.659
O5	−0.686	−0.687	−0.711
**4-hydroxy-3-methoxymandelic acid**			
C1	−0.082	−0.083	−0.077
C2	−0.212	−0.213	−0.207
C3	0.264	0.263	0.273
C4	0.282	0.282	0.277
C5	−0.262	−0.262	−0.265
C6	−0.190	−0.190	−0.187
C7	0.032	0.032	0.039
C8	0.809	0.810	0.799
H2	0.223	0.224	0.219
H5	0.219	0.220	0.201
H6	0.221	0.221	0.222
H7	0.211	0.212	0.200
H8	0.486	0.487	0.480
H9	0.505	0.506	0.487
H10	0.188	0.188	0.183
H12	0.490	0.491	0.470
H13	0.171	0.171	0.162
H14	0.180	0.179	0.182
O1	−0.751	−0.752	−0.731
O2	−0.670	−0.671	−0.670
O3	−0.638	−0.639	−0.515
O4	−0.687	−0.687	−0.676
O5	−0.589	−0.591	−0.569

* Atoms numbers as Figure 3.

**Table 6:** Wavenumbers and intensities of selected bands in mandelic acid and its derivatives spectra.

Mandelic Acid					3-Hydroxymandelic Acid					3,4-Dihydroxymandelic Acid					4-Hydroxy-3-Methoxymandelic acid					Assignment	
IR_KBr_	IR_ATR_	Raman	Theor. (1)		IR_KBr_	IR_ATR_	Raman	Theor.		IR_KBr_	IR_ATR_	Raman	Theor.		IR_KBr_	IR_ATR_	Raman	Theor.			
cm^−1^ (int.)	cm^−1^ (int.)	cm^−1^ (int.)	cm^−1^	Int.	cm^−1^ (int.)	cm^−1^ (int.)	cm^−1^ (int.)	cm^−1^	Int.	cm^−1^ (int.)	cm^−1^ (int.)	cm^−1^ (int.)	cm^−1^	Int.	cm^−1^ (int.)	cm^−1^ (int.)	cm^−1^ (int.)	cm^−1^	Int.		[45]
										3420 s	3408 m	3427 w	3850	93.8	3402 s			3850	93.8	νOH_ar_	
					3338 s	3327 m		3834	62.7	3335 s	3330 m		3792	113.0						νOH_ar_	
3400 s	3401 m		3755	86.1				3755	88.0				3756	86.1	3353 vs	3329 m		3756	89.4	νOH	
			3734	109.5				3730	112.2				3732	112.3				3728	78.9	νOH	
3070 m	3074 w	3064 vs	3197	5.4	3062 vw	3066 vw	3070 s	3198	5.8		3198 w	3175 w	3209	1.1	3087 vw		3069 vs	3203	1.7	ν(CH)	2
3031 m	3038 w	3049 m	3188	16.96	3035 w	3032 vw		3185	9.4		3032 w	3031 s	3186	1.6	3034 vw		3034 s	3182	2.9	ν(CH)	20a
2967 m		2972 m	3177	19.9				3173	6.3						2974 w	2973 w	2974 m			ν(CH)	20b
2927 m	2936 w		3167	2.1				3170	5.2	2945 w	2911 w	2916 m	3154	14.4	2935 w	2932 w	2935 s	3146	18.6	ν(CH)	7b
2716 m	2722 m		3015	17.4	2628 m	2622 w		3016	16.5				3017	18.1				3014	38.5	νCH	
1716 vs	1711 vs	1719 m	1797	335.6	1715 vs	1713 vs		1796	333.4	1708 vs 1695 vs	1692 vs	1648 m	1795	336.2	1743 vs 1718 s	1743 s 1715 s	1716 m	1796	317.3	νC=O	
		1603 m	1642	4.2	1605 s	1603 s	1609 m	1644	31.1	1622 m	1620 w	1618 s	1659	8.0	1611 m	1610 m	1609 s	1647	22.7	ν(CC)	8a
1588 w		1588 w	1627	0.4				1641	86.1	1606 s	1603 m	1605 s	1646	46.7				1634	22.9	ν(CC)	8b
1497 w	1497 w		1524	10.5	1466 vs	1465 s		1529	12.0	1537 s	1534 m	1530 vw	1544	183.7	1517 vs	1515 s		1548	225.6	ν(CC)	19a
															1460 sh	1460 sh	1461 m	1510	8.9	δ_as_(CH_3_)	
															1451 m			1488	6.7	δ_as_(CH_3_)	
1452 m	1453 m		1483	9.2				1485	96.8	1452 m	1450 m	1449 vw	1491	2.2	1439 s	1437 s	1447 m	1481	6.7	ν(CC)	19b
1378 m	1377 m		1423	18.3	1420 m	1420 m		1423	19.5	1431 s	1428 s	1414 vw	1426	18.9						βOH; δCHOH	
			1366	0.1				1368	18.8			1377 sh	1389	14.8	1380 m	1377 m	1376 w			ν(CC)	3
			1348	4.0	1359 w			1348	33.1				1351	85.4						ν(CC); βCH	14
1299 s	1296 s	1295 w	1340	92.0	1268 vs	1265 s	1265 w	1342	77.8	1350 s	1347 s	1355 m	1341	88.8	1365 sh					βOH; νC–OH	
1253 w	1253 vw	1256 vw	1307	2.5	1249 vs	1245 vs		1324	7.1	1283 s	1280 s	1293 m	1319	25.0	1270 vs	1267 s	1265 m			τCHOH(CH_2_); β(CH)	
													1303	237.0				1305	203.3	νC–OH; α(CCC); νC–CH_3_	
1229 m	1228 m	1222 vw	1253	27.5		1232 sh		1254	37.4	1259 s	1256 s	1261 w	1251	54.2	1237 vs	1234 s		1251	55.7	ωCHOH(CH_2_)	
1192 m	1192 m	1192 m	1207	6.4						1214 vs	1208 vs				1220 s	1219 vs	1193 m			β(CH); βOH_ar_	9a
1156 vw	1154 vw	1155 w	1195	8.7	1168 s	1167 m	1167 w	1196	47.2	1151 s	1148 s	1155 m	1208	43.3	1150 vs	1148 vs	1147 w	1207	27.5	β(CH); ρ(CH_3_)	9b
			1170	166.8				1179	39.5				1180	75.2				1185	48.4	βOH	
															1132 m	1131 s		1171	5.0	ρ(CH_3_)	
1062 s	1062 s	1058 vw	1089	79.7	1083 s	1079 vs	1082 m	1085	66.0	1119 s	1116 s	1111 m	1102	86.3	1061 s	1057 s	1061 m	1101	116.1	β(CH)	18a
1028 w	1030 w	1030 m	1048	7.1						1089 vs	1084 vs	1085 w			1032 s	1031 s	1032 m			β(CH)	18b
			1018	0.6				1013	4.8				960	18.2						α(CCC)	13
															954 w	954 w	953 w	1040	89.4	νO–(CH_3_)	
1004 w	1004 w	1004 s	1118	104.8	1001 m	1001 w	1000 vs	1118	83.1	982 m	980 m		1124	208.4						α(CCC); νC–OH	12
940 m	940 m		985	0.1	869 s			982	0.1	920 sh	923 sh		937	1.9		924 w	913 m	947	0.3	γ(CH)	17a
889 m	887 m		892	22.1	932 m	967 w		919	16.2	881 s	880 s	908 m	911	23.4	880 m			936	12.6	νC–COOH; α(CCC); γOH	
855 w	854 w	858 w	869	2.0	826 m	826 m	821 w	860	6.6	868 m	867 m		869	8.3	863 m	861 s		878	7.8	βC=O	
								816	1.7				836	18.2				835	20.1	γC=O; α(CCC)	
768 w	768 w	768 vw	763	16.7	732 s	731 vs	725 m	788	28.5	835 m	833 m		792	36.6	825 m	822 s	824 w	811	28.1	γ(CH)	11
733 s	731 s	732 w	727	50.9	697 s	695 s		720	54.8	807 s	801 s	784 vs	725	1.8	775 m	773 s	777 m	746	1.4	φ(CC)	4
697 s	697 s		709	33.2	674 m	671 s		695	26.7	732 m	731 m	718 m	706	36.1	732 m	731 m		707	31.8	α(CCC)	1
609 m	608 m	617 w	660	35.1			637 w	660	39.8	646 m	662 m		661	43.8	707 m	693 s	701 m	662	27.0	γC=O; βOH	
528 m			570	52.2	505 m			545	20.4	603 w		585 w	549	28.8	634 w			546	19.8	γOH	
494 m		501 w	498	4.1	462 m			475	14.4	519 m			475	20.3	533 w			471	21.8	φ(CC); γOH	16b
467 w			413	4.4				413	53.2	468 m			403	48.3	465 w			402	35.2	φ(CC); βOH	16a

s—strong; m—medium; w—weak; v—very; sh—shoulder; ν: stretching; β: in-plane deformations; γ: out of plane deformations; δ: scissoring; α: the aromatic ring in-plane bending modes; φ: the aromatic ring out-of-plane ones; τ—bending off the plane-twisting; ω—bending off the plane-fan; ρ—bending in the plane-swinging. Fundamental modes of the phenyl ring are numbered according to Varsányi [45].

**Table 7:** Experimental and theoretical chemical shifts ƍ [ppm] for mandelic acid and its hydroxy and methoxy derivatives.

Compound								
	Mandelic Acid		3-OH-Mandelic Acid		3,4-Dihydoxymandelic Acid		4-Hydroxy-3-Methoxymandelic Acid	
^13^C NMR								
assignment	Calc.	Exp.	Calc.	Exp.	Calc.	Exp.	Calc.	Exp.
C1 *	146.02	140.39	147.49	141.62	138.31	131.10	137.91	131.13
C2	129.71	126.85	113.89	113.47	118.61	114.14	128.73	110.80
C3	133.36	128.34	163.51	157.13	150.89	144.90	153.63	146.12
C4	133.39	127.87	118.26	114.51	149.05	144.90	156.53	147.26
C5	133.15	128.34	134.76	129.00	117.19	115.06	119.51	115.01
C6	133.58	126.85	124.10	117.31	120.68	117.83	125.42	119.33
C7	75.56	72.63	75.08	72.33	74.74	72.13	74.81	72.21
C8	182.50	174.36	182.60	174.04	182.56	174.48	182.73	174.39
C9	-	-	-	-	-	-	60.07	55.56
^1^H NMR								
H2	7.89	7.45	7.17	6.82	7.25	6.80	7.35	6.96
H3	7.69	7.35	--	-	-	-	-	-
H4	7.62	7.34	7.23	7.11	-	-	-	-
H5	7.61	7.35	6.98	7.11	6.95	6.64	6.90	6.72
H6	7.75	7.45	7.47	7.13	7.25	6.65	7.45	6.95
H7	3.34	5.08	3.17	4.89	3.14	4.80	3.15	4.88
H8	5.47	5.08	5.46	6.81	5.33	5.55	5.33	5.69
H9	6.60	12.69	6.05	12.41	6.55	12.38	6.51	12.44
H10	-	-	4.57	9.36	4.54	8.90	-	-
H11	-	-	-	-	5.33	8.82	4.53	8.93
H12	-	-	-	-	-	-	3.92	3.74
H13	-	-	-	-	-	-	3.44	3.74
H14							3.15	3.74

* Atoms numbers as Figure 3.

## Data Availability

Not applicable.

## Abbreviations

ABTS	2,2-azino-bis(3-ethylbenzothiazoline-6-sulfonic acid
DPPH	2,2-diphenyl-1-picrylhydrazyl radical
BDE	bond dissociation energy
CUPRAC	Cupric ion reducing antioxidant capacity
DFT	density functional theory
ETE	electron transfer enthalpy
FRAP	ferric reducing ability of plasma
HAT	hydrogen atom transfer
IP	ionization potential
MA	Mandelic acid
PA	proton affinity
PCET	proton-coupled electron transfer
SAMMA	mandelic acid condensation polymer
SPLET	sequential proton loss electron transfer
3OH-MA	3-hydroxymandelic acid
3,4-diOH-MA	3,4-dihydroxymandelic acid
4OH-3OCH_3_-MA	4-hydroxy-3-methoxymandelic acid
PDE	proton dissociation enthalpy
HOMA	harmonic oscillator model of aromaticity
HOMO	highest Occupied Molecular Orbital
LUMO	lowest Occupied Molecular Orbital
